# Supplementary material for: Communicating with Patients about COVID-19 Vaccination: A Qualitative Study on Vaccinators in Tuscany Region, Italy
Source: Vaccines (Basel). 2023 Jan 19;11(2):223. doi: 10.3390/vaccines11020223 (PMC9968224; doi:10.3390/vaccines11020223)
Supplement: Supplementary file 1 [file vaccines-11-00223-s001.zip › vaccines-2148952-supplementary.pdf]

Supplementary materials

Table S1. Characteristics of the vaccinators at individual level

Vaccinator	Age (years)	Sex	Number of patients	Average number of vaccinations administered	Previous experience with other vaccination campaign	Urbanization level	Type of vaccinators
1	29	Male	NA	5,000- 10,000	NO	Suburban/ rural	HUB
2	29	Male	NA	2,000-5,000	NO	Urban	HUB
3	29	Male	NA	2,000-5,000	NO	Suburban/ rural	HUB
4	29	Male	NA	1,000-2,000	NO	Urban	HUB
5	67	Male	NA	>10,000	YES	Suburban/ rural	HUB
6	69	Male	NA	>10,000	YES	Suburban/ rural	HUB
7	31	Female	NA	>10,000	NO	Urban	Hospital
8	71	Male	NA	5,000- 10,000	YES	Suburban/ rural	HUB
9	32	Male	NA	1,000-2,000	NO	Urban	Hospital and HUB
10	31	Female	NA	2,000-5,000	NO	Suburban/ rural	HUB
11	31	Female	NA	2,000-5,000	NO	Suburban/ rural	HUB
12	29	Male	NA	2,000-5,000	YES	Urban	USCA
13	44	Female	NA	5,000- 10,000	NO	Urban	USCA
14	28	Female	NA	1,000-2,000	NO	Urban	USCA

15	25	Female	NA	5,000- 10,000	NO	Suburban/ rural	HUB
16	55	Male	NA	2,000-5,000	YES	Urban	HUB
17	36	Male	1,600	1,000-2,000	YES	Suburban/ rural	general practitioners
18	39	Male	1,678	1,000-2,000	YES	Suburban/ rural	general practitioners
19	36	Male	1,560	1,000-2,000	YES	Suburban/ rural	general practitioners and USCA
20	34	Female	1,260	1,000-2,000	YES	Suburban/ rural	general practitioners
21	36	Female	1,450	1,000-2,000	YES	Suburban/ rural	general practitioners
22	43	Female	1,440	1,000-2,000	YES	Suburban/ rural	general practitioners
23	57	Male	1,574	1,000-2,000	YES	Suburban/ rural	general practitioners
24	69	Female	1,580	1,000-2,000	YES	Suburban/ rural	general practitioners
25	44	Male	NA	1,000-2,000	YES	Urban	USCA
26	65	Female	NA	1,000-2,000	YES	Urban	Hospital
27	38	Female	1,267	1,000-2,000	YES	Suburban/ rural	general practitioners
28	39	Female	1,200	1,000-2,000	YES	Suburban/ rural	general practitioners
29	44	Female	1,500	1,000-2,000	YES	Suburban/ rural	general practitioners
30	39	Male	760	1,000-2,000	YES	Suburban/ rural	general practitioners and USCA
